# Understanding Stress Response to High-Arsenic Gold-Bearing Sulfide Concentrate in Extremely Metal-Resistant Acidophile *Sulfobacillus thermotolerans*

**DOI:** 10.3390/microorganisms8071076

**Published:** 2020-07-19

**Authors:** Anna Panyushkina, Daria Matyushkina, Olga Pobeguts

**Affiliations:** 1 Winogradsky Institute of Microbiology, Research Centre of Biotechnology of the Russian Academy of Sciences, Leninsky Ave., 33, bld. 2, Moscow 119071, Russia; 2 Federal Research and Clinical Center of Physical-Chemical Medicine of Federal Medical Biological Agency, Malaya Pirogovskaya, 1a, Moscow 119435, Russia; d.matyushkina@gmail.com (D.M.); nikitishena@mail.ru (O.P.)

**Keywords:** acidophiles, *Sulfobacillus thermotolerans*, sulfide concentrate, arsenic, resistance, differential proteomics, cellular element content

## Abstract

Biooxidation of gold-bearing arsenopyrite concentrates, using acidophilic microbial communities, is among the largest commercial biohydrometallurgical processes. However, molecular mechanisms of microbial responses to sulfide raw materials have not been widely studied. The goal of this research was to gain insight into the defense strategies of the acidophilic bacterium *Sulfobacillus thermotolerans*, which dominates microbial communities functioning in industrial biooxidation processes at >35 °C, against the toxic effect of the high-arsenic gold-bearing sulfide concentrate. In addition to extreme metal resistance, this acidophile proved to be one of the most As-tolerant microorganisms. Comparative proteomic analysis indicated that 30 out of 33 differentially expressed proteins were upregulated in response to the ore concentrate, while the synthesis level of the functional proteins required for cell survival was not negatively affected. Despite a high level of cellular metal(loid) accumulation, no specific metal(loid)-resistant systems were regulated. Instead, several proteins involved in the metabolic pathways and stress response, including MBL fold metallo-hydrolase, sulfide:quinone oxidoreductase, and GroEL chaperonin, may play crucial roles in resistance to the sulfide ore concentrate and arsenic, in particular. This study provides the first data on the microbial responses to sulfide ore concentrates and advances our understanding of defense mechanisms against toxic compounds in acidophiles.

## 1. Introduction

Microbial biomining has been successfully used in industrial operations for decades. Natural and man-made habitats of biomining acidophilic microorganisms include sulfide ore deposits, geothermal sites, metal and coal mines with acid mine drainage, sulfate soils, pit lakes, mine heaps, and waste rock dumps [1]. Due to the ability of acidophilic chemolithotrophic microorganisms (ACMs) to oxidize ferrous iron, elemental sulfur, reduced sulfur compounds, and metal sulfides, numerous biohydrometallurgical approaches, using ACM communities, have been developed [2]. These biotechnologies are applied worldwide to recover valuable metals from the ores and ore concentrates, as well as metallurgical slags and other waste materials. Several biotechnologies (BIOX^®^, BioCOP^®^, BioNIC^®^, etc.) have been commercialized [1].

Gram-positive mixotrophic bacteria of the genus *Sulfobacillus* (*Sb.*) predominate in ACM communities in natural habitats and industrial processes of bioleaching/biooxidation of sulfide ores and ore concentrates. Due to the unique ability to oxidize all mineral substrates listed above (in the presence of small amounts of organic matter), these bacteria are widely used for the recovery of precious and other nonferrous metals at temperatures > 35 °C [2,3,4]. One of the species, *Sb. thermotolerans*, has been found to dominate different ACM communities during bioleaching of sulfide raw materials containing gold, silver, zinc, and some other nonferrous metals at 35–45 °C [5,6,7,8,9,10]. *Sb. thermotolerans* is continuously identified as the predominant bacterium in the biomining microbial communities during laboratory-scale and industrial processes of bioleaching/biooxidation of refractory gold ores [6,11,12,13]. This thermotolerant species possesses a versatile metabolism, which provides for its functioning under fluctuating conditions of natural environments and technological processes (changes in pH and temperature, pulp density, concentrations of heavy metals and metalloids, as well as the availability of energy substrates and oxygen or other electron acceptors to cells) [8,14,15,16].

In industrial operations, components of sulfide concentrates (heavy metals and metalloids) can be accumulated to high levels in bioleaching tanks. Therefore, the microbial step of ore processing requires communities of acidophiles that are resistant to high pulp densities and increased contents of metal(loid)s in the liquid phase. Biooxidation of gold-bearing arsenopyrite concentrates, using ACM communities, is among the largest commercial processes. Most arsenopyrite biooxidation processes operate at 40 °C and are dominated by communities of the sulfur- and iron-oxidizing microorganisms [17]. Some chemolithotrophs, including *Sulfobacillus* and *Acidithiobacillus* (*At.*) *ferrooxidans* strains, are tolerant to high ambient concentrations of heavy metals, which are toxic to the majority of other microorganisms at significantly lower (1–2 orders of magnitude) values; *Sb. thermotolerans* proved to be resistant to at least 765 mM Zn^2+^ and 30 mM Cu^2+^ [16].

Most studies of the heavy metal resistance mechanisms in acidophiles, including proteomic research, have focused on *At. ferrooxidans* [18,19,20,21,22,23,24,25] and some other acidophilic bacteria and archaea: *At. thiooxidans*, *At. caldus*, *At. ferrivorans*, *Leptospirillum (L.) ferrooxidans* and *L. ferriphilum*, *Ferroplasma (F.) acidarmanus*, *Sulfolobus (Sl.) metallicus* and *Sl. sulfotaricus*, and *Acidimicrobium (Am.) ferrooxidans* [26,27,28,29,30]. Analysis of the genomes of acidophiles has revealed metal resistance determinants varying in content and quantity. These microorganisms harbor metal resistance systems that are responsible for the import and efflux of heavy metal ions, as well as their extra- and intracellular sequestration and transformation to less toxic compounds [16,18,19,27,29,31]. Although complexation of metals in acidic media partially explains the phenomenon of extreme metal resistance in acidophiles [32], the differing levels of metal tolerance in acidophiles under similar growth conditions imply the involvement of other mechanisms for high metal resistance.

During the treatment of arsenic gold-bearing sulfide ores, large quantities of arsenic are released into continuous-flow aeration tanks, in which biooxidation takes place [17]. Research into the arsenic tolerance of biomining microorganisms has indicated that the acquisition of additional arsenic resistance determinants by some *At. caldus* and *L. ferriphilum* strains may be associated with their improved growth under arsenic stress [31]. The proteomic response of *L. ferriphilum* to arsenic seems to involve the arsenic resistance system, phosphate regulation, and glutathione synthesis [33]. According to the differential gene expression, the arsenic efflux system is an important pathway of arsenite detoxification in *L. ferriphilum* and *At. thiooxidans* strains [34].

A number of works have been devoted to the mechanisms of oxidation of some pure metal sulfides. The protein expression during *At. ferrooxidans* growth on the media containing pyrite, bornite, or chalcopyrite has been studied [35,36,37]. While the response of *At. ferrooxidans* to bornite exposure involved 13 proteins, mainly related to energy metabolism, detoxification, and protein synthesis, chalcopyrite contact did not lead to a significant alteration in the level of protein expression [36]. In another *At. ferrooxidans* strain, a small decrease in the *rus* operon gene expression was observed in the presence of chalcopyrite, while the presence of covellite caused a remarkable decrease in the expression of these genes [38]. Adaptations of *L. ferriphilum* to growth on chalcopyrite include the possibly enhanced production of reducing power, reduced carbon dioxide fixation, enhanced chemotaxis and motility, as well as elevated levels of RNA transcripts and proteins involved in heavy metal resistance, with special emphasis on copper efflux systems [39]. The gene expression analysis has indicated that *Acidithiobacillus* sp. FJ2 can survive and leach uranium under the stress conditions of different uranium ore pulp densities (up to 50%) by modulation in the *rus* operon gene responses, increasing the expression levels of the *cyc2*, *cyc1*, *rus*, and *coxB* genes [25]. Different research groups have studied the proteomic changes that are involved in the biofilm formation by two Gram-negative acidophilic bacteria: *At. ferrooxidans* [37,40,41] and *Leptospirillum* spp. [42,43,44]. The formation of biofilms is considered to be one of the central aspects of the bioleaching of sulfide ores and ore concentrates.

Although numerous works have been devoted to the interactions of acidophilic microorganisms with metal(loid)s and some pure metal sulfides, little is known about the molecular mechanisms of microbial responses to sulfide raw materials, including arsenic-containing sulfide concentrates. To our knowledge, no data on the molecular responses of chemolithotrophic acidophiles to sulfide concentrates containing nonferrous (including precious) metals, and, particularly, arsenopyrite concentrates of gold-bearing sulfide ores, have been reported. At the same time, biotechnologies for processing of complex sulfide raw materials are widely applied, and studies of the mechanisms of responses of biotechnological microbial cultures to arsenic-rich sulfide concentrates are of interest and importance for the optimization of bioleaching operations. The goal of this study was to gain insight into the defense strategies of *Sb. thermotolerans* against toxic effects of the high-arsenic gold-bearing sulfide concentrate. The research objectives were to investigate its effect on the growth and oxidative activity of *Sb. thermotolerans*, proteome reorganization, and cellular accumulation of metal(loid)s.

## 2. Materials and Methods

### 2.1. Materials

The flotation concentrate of the gold-bearing pyrite-arsenopyrite sulfide ore was used in the experiments. The mineral composition of the ore concentrate was as follows: FeS_2_ (48 wt%), FeAsS (35 wt%), ZnS, CuFeS_2_, PbS, Au (135.2 g/t), and Ag (160 g/t). The contents of sulfidic sulfur (S_S_) and sulfidic arsenic (As_S_) in the ore concentrate were 28.1 and 16 wt%, respectively. Table 1 shows the chemical composition of the ore concentrate. The particle size distribution of the concentrate sample had a P_80_ of 44 μm.

### 2.2. Research Object and Cultivation Conditions

*Sb. thermotolerans* strain Kr1^T^ (VKM B-2339^T^ = DSM 17362^T^) that was used in this study was obtained from the Collection of Microorganisms of Winogradsky Institute of Microbiology, Russian Academy of Sciences (Moscow, Russia). The strain was cultured in 250 mL Erlenmeyer flasks (100 mL of the liquid medium) on a Unimax-1010 rotor shaker (Heidolph Instruments, Schwabach, Germany; 220 rpm) in an Inkubator-1000 thermostat (Heidolph Instruments, Schwabach, Germany). Experiments were carried out using the modified 9K medium [16] containing ferrous sulfate (48 mM Fe^2+^) as an energy source and yeast extract (0.02%, *w*/*v*). The 9K medium supplemented with 10, 20, or 30 g/L ore concentrate instead of ferrous iron was used to determine the characteristics of *Sb. thermotolerans* Kr1 growth and substrate oxidation in the presence of different amounts of the sulfide concentrate. To obtain the biomass for a subsequent proteomics analysis and determination of the cellular element content, the strain Kr1 was grown in 2500 mL Erlenmeyer flasks containing 1500 mL of the modified 9K medium and ferrous iron (48 mM Fe^2+^) or 2% (*w*/*v*) ore concentrate with mixing by air agitation (sterile air was supplied at a flow rate of 2 L min^–1^) in a Redline RI 53 incubator (Binder, Tuttlingen, Germany). In all experiments, *Sb. thermotolerans* Kr1 was cultured at 39 ± 1 °C. The initial ambient pH value was adjusted to 1.7 with 10 N H_2_SO_4_. The amount of inoculum was 10% (*v*/*v*).

### 2.3. Cell Treatment and Pellet Lysis

For subsequent proteomics analysis and determination of cellular elements, late-exponential cells of *Sb. thermotolerans* Kr1 were collected by centrifugation (10,000× *g*, 15 min, 4 °C) and treated as further described below. To assess the total cellular and intracellular element accumulation, two types of *Sb. thermotolerans* samples were used. The first one (for the determination of the total cellular concentrations of elements) contained intact cells that were washed in the acidified salt base of the iron-free 9K medium. Cells were further pelleted by centrifugation (10,000× *g*, 15 min, 4 °C) and dried overnight at 80 °C to calculate the dry weight of the cell pellet. The second variant (determination of the intracellular element concentration) contained cell-free lysates that were obtained after sonication of *Sb. thermotolerans* cells using a UZDN-2T ultrasonic disintegrator (Akadempribor, Sumy, Ukraine) (1.5 min with 2-min intervals for cooling, four times; 22 Hz, 40 mA) and subsequent centrifugation (40,000× *g*, 30 min, 4 °C) to pellet cell debris. Prior to the ultrasound disintegration, metals were desorbed from cell walls. To achieve this, cells were rinsed with acidified distilled water (three times), centrifuged, suspended in a 50 mM Tris-HCl buffer (pH 6.8) containing 1 mM EDTA (ethylenedinitrilotetraacetic acid) (10 min), and washed from EDTA with the latter buffer. Lysates were air-dried to concentrate cellular contents for subsequent determination of elements by analytical techniques (Section 2.6). For proteomics studies, cell pellets were washed twice with the salt base of the 9K medium (pH 1.8) containing no energy substrates, washed with a 50 mM Tris-HCl buffer (pH 6.8), and suspended in the latter. The suspension in the buffer was supplemented with 1 mM EDTA and 1 mM PMSF (phenylmethylsulfonyl fluoride; Sigma-Aldrich, Steinheim, Germany) and stored at −70 °C prior to the proteomic analysis.

### 2.4. Proteomic Analysis

#### 2.4.1. Two-Dimensional Difference Gel Electrophoresis (2D DIGE)

Prior to the 2D DIGE, *Sb. thermotolerans* cell pellets were treated with a 5-μL mixture of 30% CHAPS (3-[(3-Cholamidopropyl)dimethylammonio]-1-propanesulfonate) and 10% NP40 detergents (Sigma-Aldrich, Steinheim, Germany) and a 1-μL mixture of nucleases (Nuclease Mix, GE Healthcare, Amersham, UK). The cell pellets were incubated for 30 min at 4 °C and dissolved in the buffer for isoelectric focusing (40 mM Tris-HCl, PH 9.5), containing the following: 8 M urea, 2 M thiourea, 4% CHAPS, and 2% NP40. The samples were centrifuged at 14,000× *g* for 15 min. Protein concentration in the samples was measured by the Bradford method using the Quick Start Bradford dye (Bio-Rad, Hercules, CA, USA). The sample proteins were labeled with Cy3 (green) or Cy5 (red) CyDye DIGE Fluor minimal dyes (Amersham Biosciences, Vienna, Austria) according to the manufacturer’s recommendations (400 pmol per 50 μg protein). The binding reaction of cyanines with protein was stopped by adding a 10 mM lysine solution. DTT (dithiothreitol; to the final concentration of 100 mM) and Ampholine 3–10 (to 1%) (Bio-Rad, Hercules, CA, USA) were subsequently added. Prior to mixing a pair of the samples compared, we performed an electrophoretic separation of each of them by 12% *PAGE* under denaturing conditions. After electrophoresis, the gel was scanned on the TyphoonTrio scanner (Amersham Biosciences, Vienna, Austria) at a laser wavelength of 532 nm (green fluorescence) and 633 nm (red fluorescence). The value of the total fluorescence intensity was detected for each sample. Given these values, the two samples labeled with a different cyanine were mixed in a certain ratio, based on the overall alignment of the fluorescence intensity values for each of them. Isoelectrofocusing was performed in 18-cm glass tubes in 4% polyacrylamide gel of the following composition: 8 M urea, 2% ampholines (pH 3–10), 4% ampholines (pH 5–8), 6% solution containing 30% CHAPS and 10% NP40, 0.1% TEMED (N,N,N′,N′-tetramethylethane-1,2-diamine), and 0.02% ammonium persulfate. The total protein content was 200–250 μg per tube. Isoelectrofocusing was performed in the following mode: 100, 200, 300, 400, 500, and 600 V (45 min); 700 V (10 h); and 900 V (1 h). On completion of isoelectrofocusing, the tubes were equilibrated (for 30 min) in the buffer containing the following: 6 M urea, 30% glycerol, 62.5 mM Tris-HCl (pH 6.8), 2% SDS, 20 mM DTT, and bromophenol blue. The tubes were subsequently transferred to the surface of the gradient polyacrylamide gel (9–16%) and fixed with 0.9% agarose using bromophenol blue. Electrophoresis was performed in a Tris-glycine buffer under the following conditions: 20 mA on glass (20 min), 40 mA on glass (2 h), and 35 mA on glass (2.5 h) with chamber cooling to 10 °C. The results were analyzed by the PDQuest 8.0 software (Bio-Rad, Hercules, CA, USA). For spot excision, the gels were silver stained as described [45].

#### 2.4.2. Trypsin Digestion and MALDI Mass Spectrometry

The protein spots (after 2D DIGE) were subjected to trypsin in-gel hydrolysis as described [46]. Gel pieces of 2 mm^3^ were excised and washed with 10 μL of a 15 mM sodium thiosulfate and 50 mM potassium hexacyanoferrate (III) mixture for 10 min at room temperature, washed twice with deionized water, dehydrated with 100 μL of acetonitrile, and air-dried. The gel pieces were subsequently treated with 3 μL of a 15 mg/mL trypsin solution (Promega, Madison, WI, USA) in 50 mM ammonium bicarbonate for 16 h at 37 °C. Peptides were extracted with a 0.5% trifluoroacetic acid water solution (6 μL) for 30 min.

Aliquots (2 μL) of the sample were mixed on a ground steel target with a 0.5 μL of 2,5-dihydroxybenzoic acid (Sigma-Aldrich, Steinheim, Germany) solution (30 mg/mL in 30% acetonitrile/ 0.5% trifluoroacetic acid), and the droplet was left to dry at room temperature. Mass spectra were recorded on an Ultraflex II MALDI-ToF-ToF mass spectrometer (Bruker Daltonik, Bremen, Germany) equipped with Nd laser. The [M + H]^+^ molecular ions were measured in a reflector mode; the accuracy of mass peak measurement was 70 ppm.

#### 2.4.3. Protein Identification

Proteins were identified by the peptide fingerprint search with Mascot software (Matrix Science Inc., Boston, MA, USA) through the *Sb. thermotolerans* Kr1 protein database (accession number CP019454; https://www.ncbi.nlm.nih.gov/nuccore/CP019454.1?report=genbank). Protein scores greater than 45 were considered significant (*p* < 0.05). The quantitative assessment was performed with the PDQuest 8.0 software (Bio-Rad, Hercules, CA, USA). Differentially expressed proteins with a fold change of ≥2.0 were discussed.

### 2.5. Protein Characterization and Pathway Mapping

Functional characterization of the proteins of interest, encoded by the genome of *Sb. thermotolerans* Kr1, was carried out using the KOALA (KEGG Orthology and Links Annotation) system [47]. Pathway mapping was carried out using tools of the KEGG software package.

### 2.6. Analytical Techniques

The pH value and concentrations of Fe^3+^ and Fe^2+^ were measured as previously described [16]. Arsenic in the liquid phase was determined by iodometric titration involving the binding of iron ions with TiCl_3_ as reported [48]. The concentrations of zinc and copper in the growth media were measured using a Perkin Elmer 3100 flame atomization atomic absorption spectrometer (PerkinElmer, Waltham, MA, USA). The chemical composition of the concentrate (iron, arsenic, and other elements) was determined using an inductively coupled plasma atomic emission spectrometer (Optima-4300 DV, PerkinElmer, Waltham, MA, USA) according to the manufacturer’s recommendations. The sulfur content was measured by the gravimetric analysis [10]. The sulfate concentration was measured turbidimetrically as described [8]. The mineralogical composition of the sulfide concentrate was determined by X-ray diffraction (PANalytical X’Pert PRO MPD, Almelo, The Netherlands). The sulfidic mineral contents were adjusted according to the element contents.

In cell samples, zinc, copper, lead, gold, silver, antimony, arsenic, and iron were measured using inductively coupled plasma mass spectrometry (Elan-6100, PerkinElmer, Waltham, MA, USA) and inductively coupled plasma atomic emission spectroscopy (Optima-4300 DV, PerkinElmer, Waltham, MA, USA) according to the manufacturer’s recommendations.

The quantitative assessment of microorganisms was carried out by direct counts in the Goryaev chamber and by the method of serial terminal ten-fold dilutions using a Mikmed-2 microscope (LOMO, St. Petersburg, Russia) equipped with a phase contrast device. Microscopy was also used to determine morphological alterations within the cell population of *Sb. thermotolerans*.

### 2.7. Statistical Methods

Chemical and mineral analyses were carried out in duplicate with three replicates (ore concentrate) and in two experiments with three samples and three series of measurements for each sample (elements in cells and the liquid phase). Two series of experiments on the bacterial growth included three parallels (flasks) and three replicates for each measured parameter. Statistical processing was performed using Microsoft Excel 2010. The standard deviation (SD) of the arithmetic mean was calculated, and the significance of the results was assessed using the Student’s *t*-test at the significance level *p* ≤ 0.05. Two series of proteomic experiments under different growth conditions and three protein extractions (biological replicates) with three or four 2D gels for each extraction were carried out. Data were statistically processed using the PDQuest 8.0 software (Bio-Rad, Hercules, CA, USA). The means of three biological replicates in two experiments (±SD) were discussed.

## 3. Results and Discussion

### 3.1. Toxic Effect of Sulfide Concentrate on Bacterial Growth and Oxidative Activity

A comparison of the growth and oxidative characteristics of the *Sb. thermotolerans* Kr1 that was not preliminarily adapted to the presence of the ore concentrate (10, 20, or 30 g/L) indicated the following. An increase from 10 to 20 g/L resulted in insignificant changes in the maximum cell yields: 3.5 and 3.0 × 10^8^ cells/mL, respectively (Figure S1a,b). However, the maximum specific growth rate decreased ~3 times (*µ*_max_, 0.33 and 0.12 h^−1^ at 10 and 20 g/L, respectively), and the maximum iron oxidation rate (*V*_max_, 4.7 and 1.8 mM/h, respectively) decreased more than 2.5 times. A 10-h lag phase preceded the active growth and oxidation. According to microscopic observations, ~1–1.5% of forespores (an intermediate stage between a vegetative cell and a dormant spore) were present in the cell population. During 117 h of cultivation, the pH values varied within 1.7–1.9. At all points of the curve, zero Fe^2+^ concentration was detected, indicating the rapid oxidation of the leached ferrous iron to ferric iron. Overall, changes in the growth parameters, which were observed in the presence of 20 g/L of the sulfide concentrate, indicated partial inhibition of microbial activity (toxic effect on microbial cells) by the components of the ore concentrate. We observed a significant suppression of the growth of the nonadapted *Sb. thermotolerans* Kr1 culture in response to 30 g/L of the ore concentrate in the medium (*µ*_max_, 0.02 h^−1^; *V*_max_, 0.5 mM/h) (Figure S1c).

Based on these results, we selected two variants for subsequent studies of the mechanisms of resistance to the arsenopyrite concentrate. *Sb. thermotolerans* Kr1 was grown in the presence of 20 g/L sulfide concentrate (the final growth and oxidation parameters were 2.2 × 10^8^ cells/mL and 39 mM Fe^3+^, respectively; Figure S1b) and under the optimal growth conditions (control) when ferrous iron was used as an energy substrate (final parameters were 3.0–3.1 × 10^8^ cells/mL and 43 mM Fe^3^^+^; Figure S1d). As mentioned above, the ore concentrate (20 g/L) had a certain toxic effect on the growth of *Sb. thermotolerans* Kr1. The latter, however, was able to adapt to its presence during cultivation. Due to the sufficient bacterial growth under these stressful conditions, it was, therefore, possible to study the proteomic response of the strain and to analyze the cellular contents of metal(loid)s.

### 3.2. Metal Accumulation by Cells

The sulfide concentrate used in this study was characterized by a high content of As (19.1 wt%), as well as the presence of Zn, which also contributed to the toxic effect on the bacterial cells. During biooxidation of the sulfide ore concentrate, ferrous iron, arsenic, and zinc were gradually released from the solids into the medium (Table 1). The cellular contents of several metal(loid)s accumulated by the late-exponential *Sb. thermotolerans* cells significantly increased compared to those under the optimal growth conditions (Table 2).

After 80 h of growth, 1.5 mM Zn^2+^ was released (Table 1). Except for iron, the total zinc sorption by the cells and intracellular accumulation of zinc were at the highest levels among all elements; they increased 25 and 15 times, respectively, compared to the control values (Table 2). At the same time, while iron absorption was similar in both variants (0.6–0.8 mg/g dry weight), total iron accumulation was one order of magnitude lower than in the variant grown under the optimal conditions (6.5 and 60.2 mg/g dry weight, respectively). This may result from a slow rate of ferrous iron leaching from the sulfide concentrate, competition of metal ions for the binding sites on the cell surface, and specific characteristics of the cell envelope of *Sb. thermotolerans* Kr1 [8]. In the presence of Cr^6+^, Fe sorption by the biomass of the yeast *Saccharomyces (Sc.) cerevisiae*, fungus *Aspergillus (As.) niger*, and bacterium *Streptococcus (St.) equisimilis* was also partially inhibited [49]. A similar pattern was observed for iron accumulation by the *Sc. cerevisiae* stationary-phase cells in the presence of ZnCl_2_: While the cellular accumulation of Zn increased ~25 times, Fe accumulation decreased three times (from 5 to 13 and from 0.25 to 0.08 mg/g dry weight, respectively). The amount of intracellular iron decreased in a manner that was dependent on the concentration of zinc in the medium [50].

Copper accumulation was ~6–8 times higher than in the culture grown under the optimal conditions. The total and intracellular Sb content increased ~30 and 80 times, respectively. The total accumulation of Ag, Au, and Pb in the concentrate-grown cells increased at least two orders of magnitude, and the intracellular accumulation of these elements was recorded (Table 2).

Among the most interesting results of this study was the high-level arsenic resistance of *Sb. thermotolerans*. In acidic conditions, arsenic is most commonly found as either arsenate (As^5+^/AsO_4_^3−^) or arsenite (As^3+^/As(OH)_3_). Arsenate enters the cell via the phosphate uptake system and is toxic by acting as a phosphate analog, while the more toxic arsenite enters cells via aquaglyceroporins and some sugar transporters and is toxic by binding sulfhydryl groups [31]. The total As concentration in the *Sb. thermotolerans* Kr1 growth medium reached a high level of 35.3 mM (4.4 mM As^5+^ and 30.9 mM As^3+^) after 80 h of cultivation. Most arsenic was in the highly toxic arsenite (As^3+^) form. These conditions may be compared to the initial stages of processing of arsenopyrite concentrates when a microbial community gradually adapts to increasing concentrations of dissolved metal(loid)s in the liquid phase. The demonstrated growth of *Sulfobacillus* strains in bioreactors with moderate to high metals concentrations has indicated their strong ability to adapt to the presence of soluble metals in their environment [51]. In laboratory-scale and industrial operations, arsenic concentrations may vary within 8–120 mM at pulp densities of 4–20 wt%, while iron concentration can reach 250 mM [8,12,48,52]. In fact, a bioleaching microbial community, which contained *Sb. thermotolerans* and was gradually adapted to this ore concentrate at a high pulp density (20%, *w*/*v*), tolerated up to 131 As^3+^ during the long-term bioleaching process (our unpublished data). Another microbial community dominated by *Sb. thermotolerans* tolerated at least 81 mM arsenic in the course of bioleaching of a 200 g/L gold-bearing pyrite-arsenopyrite ore concentrate that contained 3.63% As_S_ in its original composition [12]. A community consisting of *Sb. thermosulfidooxidans* and *At. ferrooxidans* strains tolerated up to 57–115 mM As while oxidizing the ore concentrate of the composition that was similar to the one of the concentrate used by us (40% FeS_2_ and 35% FeAsS (16% As_S_); 134.6 g/t Au; 200 g/t Ag) at 30 and 42 °C in the pH range of 1.2–2.0 [48]. *Sulfobacillus* spp. strains were shown to tolerate up to 25–100 mM As^5+^ when pure cultures [51] and microbial communities [53] were used. Other members of acidophilic microbial communities are also known to be highly arsenic-resistant. Thus, *L. ferriphilum* strains conferred resistance to 40–60 mM As^5+^ and As^3+^, while *L. ferrooxidans* was resistant to 30 mM As^3+^ and 20 mM As^5+^ [17]. *At. ferrooxidans* and *At. caldus* strains tolerated 20–84 and 30 mM As^3+^, respectively [52,54,55].

Our study indicated that *Sb. thermotolerans* cells accumulated high amounts of As (0.9 mg/g dry weight) compared to most other microorganisms. Cellular and intracellular accumulation of As (after 80 h of cultivation) proved to be 540 and 250 times higher, respectively, than under the optimal conditions of growth. Earlier, we have identified the genes that are involved in the mechanisms of resistance to arsenic (As^3+^) in *Sb. thermotolerans* Kr1. Although the repressor gene of the arsenic resistance operon (*arsR*) is present in the *Sb. thermotolerans* Kr1 genome, no arsenate reductase (*arsC*) has been identified. At the same time, the ArsA/ArsB ATPase pump, which is predicted to export arsenite and antimonite, is encoded by the *Sb. thermotolerans* genome [16].

According to the previous studies, cells of the most arsenic-resistant bacterial strains, which can potentially be used in bioremediation, accumulate up to ~0.025 mg As per g of cell pellet [56]. The capacity of algal cells of *Synechocysis* sp. for metal accumulation is similar to *Sb. thermotolerans* cells: 0.9–1 mg/g dry weight, although the minimum inhibitory concentration value of As for *Synechocystis* sp. is only 0.5 mM As in the growth medium [57]. Nevertheless, it should be noted that the resistance of these organisms can be compared only indirectly, due to the differences in their cellular organization and conditions of growth. The most amazing examples of arsenic-resistant organisms are *Theonella swinhoei* (a common Indo-Pacific sponge) and *Entotheonella* sp. fractions from the sponge. The former has been singled as a hyperaccumulator of arsenic, with the highest As concentration (8.6 mg/g) recorded in any organism from an uncontaminated environment [58], while *Entotheonella* sp. can tolerate the absolute As maximum of 12 mg/g [59]. Our results revealed a high level of arsenic accumulation by *Sb. thermotolerans*, which, however, retained its ability for active growth and substrate oxidation.

In general, a comparison of cellular metal contents in microorganisms, especially metal-resistant chemolithotrophs, is problematic due to scarce information about metal accumulation by growing microbial cells. The biomass of *Sc. cerevisiae*, *As. niger*, and *St. equisimilis* sorbs up to 16–22 mg Fe/g dry weight, depending on the cultivation conditions [49], which is approximately 3–5 times lower than the level of Fe accumulation by *Sb. thermotolerans* growing cells. The Zn and Pb biosorption by *Delftia tsuruhatensis* (a bacterial strain resistant to metals and isolated from mine tailings) reaches 13 and 45 mg/g dry weight, respectively [60]. The cell biomass of the most studied Gram-negative acidophilic chemolithotrophic bacteria of the genus *Acidithiobacillus* sorbs high levels of heavy metals. Biosorption by *At. ferrooxidans* is up to ~83 mg Zn^2+^/g dry biomass when the initial zinc concentration in the culture medium is increased from 25 to 150 mg/L [61]. The *At. thiooxidans* biomass absorbs Zn (95–172 mg/g dry biomass depending on the temperature of cultivation) more efficiently than Cu (32–40 mg/g) [62], while *At. caldus* biomass possesses even a higher sorption capacity: Up to 110 mg/g of Zn, 304 mg/g of Cu, and 310 mg/g of Pb [63]. The goal of our experiments with metabolically active growing cells was different from the efficient sorption by cell biomass. Therefore, these values cannot be directly compared. To our knowledge, no data on metal accumulation by acidophiles under similar conditions have been reported, which makes it difficult to assess the efficiency of metal accumulation by *Sb. thermotolerans* cells. The substantial difference in the metal accumulation by the strain Kr1 that was grown under optimal and stress conditions (high-arsenic sulfide concentrate) can be noted. Overall, *Sb. thermotolerans* cells proved to accumulate high concentrations of heavy metals. Despite the high cellular level of metal(loid)s, the strain retained growth and possessed high iron-oxidizing activity. Moreover, according to the concentrations of As^3+^ released into the culture medium during cultivation and high level of cellular accumulation of arsenic, this bacterium proved to be among the most As-resistant microorganisms.

### 3.3. Proteome Reorganization in Response to the Pyrite-Arsenopyrite Concentrate

**Figure 1:**
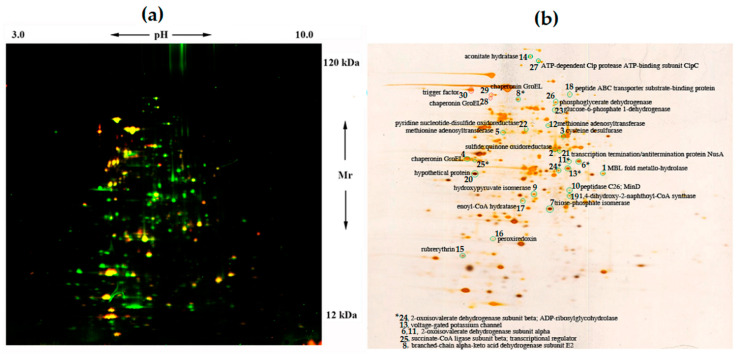
Two-dimensional (2D) DIGE analysis of *Sb. thermotolerans* cells grown in the presence of the arsenopyrite concentrate (Cy3 dye, green) and in the control medium (Cy5 dye, red) (**a**) and a corresponding silver-stained gel (**b**). Arrows (**a**) indicate pH (3–10) and Mr (12–120 kDa) ranges. Circled spots (**b**) correspond to proteins ≥2-fold up- (green circles) or downregulated (red circles) in response to the concentrate (spot numbers refer to Table 3) and identified by MALDI (Matrix-Assisted Laser Desorption/Ionization) mass spectrometry.

The data on the bioinformatics analysis of the *Sb. thermotolerans* Kr1 genome [16] and a high level of metal accumulation (this study) suggest efficient mechanisms of defense against high concentrations of heavy metals and metalloids. A comparative analysis of the protein profiles of the control variant and the cells grown in the presence of the sulfide concentrate (Figure 1a,b) was the principal study to elucidate the possible strategies of metal(loid) resistance in this bacterium.

Out of 33 proteins, which were differentially expressed in the *Sb. thermotolerans* cells in response to the sulfide concentrate, only three proteins proved to be downregulated (Figure 1, Table 3).

**Figure 2:**
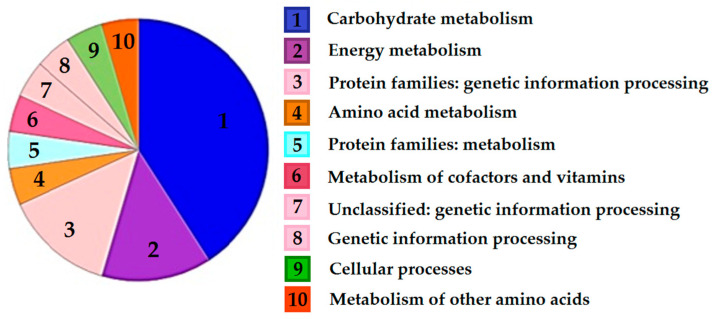
Results of the ortholog annotation of the proteins that were regulated in the *Sb. thermotolerans* cells in response to the pyrite-arsenopyrite concentrate, using KOALA (KEGG Orthology and Links Annotation) tools [47]. The pie chart shows the relative contribution of the functional categories of regulated proteins. The categories are numbered (1–10) and shown in different colors, according to KEGG color codes.

According to a functional classification in clusters of orthologous groups (COGs), all regulated proteins were grouped into ten functional categories. More than half of the differentially expressed proteins were involved in carbohydrate and energy metabolism. Other proteins were assigned to the following functional categories: Genetic information processing, amino acid and protein metabolism, metabolism of vitamins and cofactors, and cellular processes (Figure 2).

#### 3.3.1. Stress Response

The stress response of *Sb. thermotolerans* to the ore concentrate involved the regulation of the synthesis of chaperones and stress proteins. Thus, the ATP-binding ClpC subunit of the ATP-dependent Clp protease (a component of the Clp chaperone-protease complex, which is involved in protein degradation and disaggregation) was upregulated (Figure 1, Table 3). The ClpC subunit is required for growth at high temperatures (probably, it functions as a chaperone during heat shock) and for biofilm formation. It may act as a chaperone regulating CtsR activity [64] and participate in stress response to cadmium and copper ions [65]. In *At. ferrivorans* from the natural acid mine drainage environment (the Tinto River), the ClpB chaperone was one of the proteins that conferred arsenic resistance [66]. At the same time, the ribosome-associated molecular chaperone trigger factor (TF) was slightly downregulated in *Sb. thermotolerans* (2.4-fold change). It was also downregulated in *E. coli* cells grown in the presence of excess Zn^2+^ [67] and only transiently produced during the growth-arrested phase in Cd^2+^-stressed *E. coli* cells [68].

In the sample of *Sb. thermotolerans* Kr1 cells, which were grown in the medium containing the sulfide concentrate, one of the most abundant protein spots corresponded to the GroEL molecular chaperonin (5.3-fold change). The other two spots that were also identified as GroEL were slightly downregulated (2.1 times) (Figure 1, Table 3). Most likely, the observed changes in the location of the GroEL protein on the 2D map were due to the presence of post-translational modifications of this protein under conditions of the bacterial growth in the medium containing the sulfide concentrate. Previous studies have shown that the synthesis of GroEL is induced in *Bacillus cereus* cells exposed to silver ionic stress [69] and in *E. coli* cells in the presence of excess zinc in the culture medium [67]. In response to cadmium, GroEL is upregulated in *Sphingomonas* sp. [70] and Cd-tolerant *Cupriavidus taiwanensis* [71]. TF, GroEL, and one more chaperone (DnaK) have distinct but overlapping functions in assisting *de novo* folding. While the GroEL chaperone is essential under all growth conditions, TF and DnaK are not. The function of TF may be compensated by enhanced action of GroEL and DnaK [72]. In the case of *Sb. thermotolerans*, we observed the upregulation of GroEL and downregulation of TF. We, therefore, suggest the possible role of the GroEL chaperonin in the tolerance mechanisms to heavy metals in *Sb. thermotolerans*. Due to the probable modification, the GroEL chaperonin might be activated under the stressful conditions of growth.

Interestingly, the MBL fold metallo-hydrolase was the most upregulated protein (6.3 times) in response to the ore concentrate. This metal-related stress protein is highly upregulated in *Staphylococcus cohnii,* which is the probable mechanism for the survival of the organism under arsenic stress [73]. The substrate-binding component of the ABC-type thermophilic oligopeptide-binding transporter was also upregulated (2.3-fold) in *Sb. thermotolerans*. This protein belongs to the type 2 periplasmic binding fold protein superfamily, participating in the transport of different metabolites: Amino acids, carbohydrates, ions, and polyamines. ABC-transporters also carry out the co-transport of metals in complex with different ligands: Amino acids, phosphates, peptides, and organic acids [74]. Nevertheless, no regulation of the proteins belonging to the specific metal(loid) resistance systems was found in response to toxic amounts of the ore concentrate in the growth medium.

Comparative proteomics revealed also the indicators of oxidative stress in response to the pyrite-arsenopyrite concentrate. Selenocysteine-containing peroxiredoxin PrxU (the antioxidant protein that reduces and detoxifies hydrogen peroxide, peroxynitrite, and organic hydroperoxides) was upregulated in the ore-grown *Sb. thermotolerans* cells (Figure 1, Table 3). In acidophilic bacteria, the AhpCF alkyl-hydroperoxidase/peroxiredoxin couple is suggested to be involved not only in the detoxification of organic peroxides but also in the degradation of H_2_O_2_ [37]. The upregulation of the synthesis of rubrerythrin (another scavenging enzyme of the oxidative stress defense system), reducing toxic hydrogen peroxide, was also observed in *Sb. thermotolerans*. Rubrerythrin is encoded in several *Leptospirillum* spp. genomes and may function as H_2_O_2_ reductase [37].

Habitats of ACMs (for instance, acid mine drainage ecosystems) are characterized by low pH values and high contents of metal ions. Iron, copper, cobalt, nickel, and some other metals are known to increase the production of reactive oxygen species (ROS) [74]. Metal sulfides, such as pyrite and chalcopyrite, generate extracellular ROS upon exposure to acidic water. The recent study of the impact of H_2_O_2_ on *At. ferrooxidans* DSM 14882^T^ has indicated that iron- or sulfur-grown cells show a higher sensitivity toward H_2_O_2_ than pyrite-grown cells. In total, 80 proteins that are potentially involved in general stress responses (such as chaperones, heat- or cold-shock proteins, and metal resistance proteins) and specific ROS defense mechanisms are regulated in the presence of H_2_O_2_, while 30 of these proteins are enhanced in pyrite biofilm cells [37]. The synergistic effect of Li^+^ and Co^2+^, accumulated in the leachate in the simulated bioleaching system with 4.0% of LiCoO_2_ slurry, has been shown to cause the oxidative stress in the acidophilic microbial consortium including mainly *Sb. thermosulfidooxidans* and *L. ferriphilum* [75]. Although the oxidative stress results in increased intracellular ROS, the addition of the exogenous glutathione (which acts as an antioxidant) increases the activities of GSH peroxidase and catalase to scavenge the excessive intracellular ROS, and this results in improved bioleaching of LiCO_2_ by the community [75].

We have previously shown that the genome of *Sb. thermotolerans* Kr1 encodes glutathione (GSH) peroxidase, which reduces hydroperoxides by glutathione, while no other *Sulfobacillus* species harbor this enzyme. The GSH peroxidase activity of *Sb. thermotolerans* Kr1 is dependent on the aeration mode, showing higher values under conditions of intense aeration [16]. As mentioned above, arsenite (As^3+^) reached high ambient concentrations (up to 30 mM) during the *Sb. thermotolerans* growth in the presence of the ore concentrate. Although As^3+^ could potentially deplete intracellular glutathione, according to the known mechanism of its action [76], the level of the GSH peroxidase synthesis did not change in our experiments. However, similarly to an increase in the GSH peroxidase activity under oxidative stress [16], ROS could affect the activity of this antioxidant enzyme and, therefore, the intracellular pool of glutathione.

#### 3.3.2. Metabolic Reorganization

**Figure 3:**
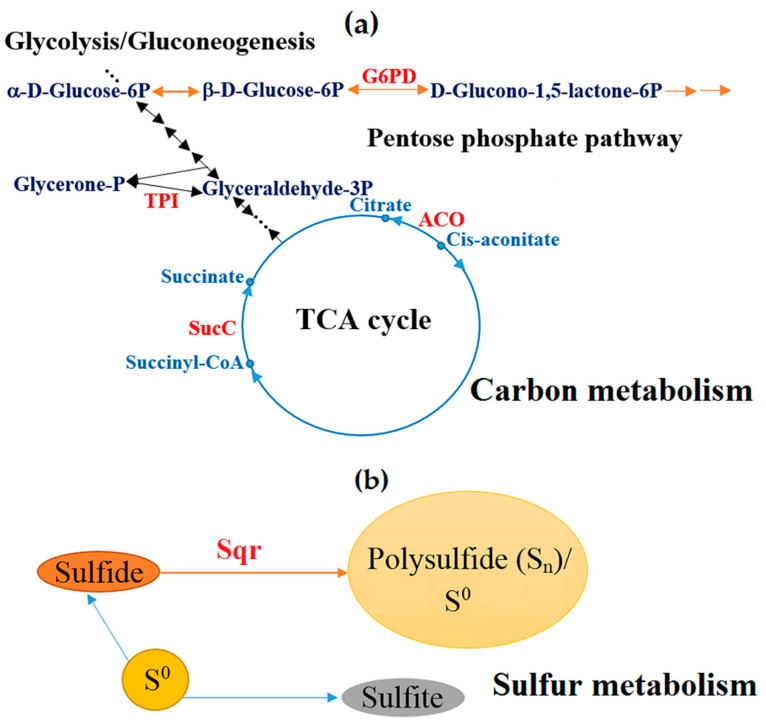
Reinforcement of (**a**) carbon and (**b**) sulfur metabolism pathways in *Sb. thermotolerans* in response to the gold-bearing pyrite-arsenopyrite concentrate. TPI: Triosephosphate isomerase [EC 5.3.1.1]; G6PD: Glucose-6-phosphate 1-dehydrogenase [EC 1.1.1.49 1.1.1.363]; ACO: Aconitate hydratase [EC 4.2.1.3]; SucC: Succinyl-CoA synthetase beta subunit [EC 6.2.1.5]; Sqr: Sulfide:quinone oxidoreductase [EC 1.8.5.4]. Substrates, intermediates, and products are indicated in blue (**a**) and upregulated enzymes are indicated in red (**a,b**).

The data obtained in this study revealed the enforcement of metabolic pathways in response to the high content of the sulfide concentrate. An increase in the biosynthesis levels of the enzymes involved in the carbohydrate and energy metabolism, amino acid biosynthesis/degradation pathways, and the TCA cycle was observed (Table 3). Figure 3a shows the upregulation of several enzymes that are involved in the TCA cycle and carbohydrate catabolism, as well as one enzyme, which is common for the Embden-Meyerhof-Parnas pathway, gluconeogenesis, and Calvin cycle.

The differential regulation of the proteins involved in energy metabolism was also found. For instance, 1,4-dihydroxy-2-naphthoyl-CoA (catalyzes the main step in menaquinone biosynthesis, electron transport) and two different FAD-dependent pyridine nucleotide-disulfide oxidoreductases were upregulated in response to the sulfide concentrate. Among all regulated proteins, an increase in the synthesis of the sulfide:quinone oxidoreductase (SQR) was one of the highest (5.8 times) (Table 3). This enzyme (eight copies in the genome of *Sb. thermotolerans* Kr1) catalyzes the transformation of hydrogen sulfide to polysulfide in the sulfur metabolism pathway (Figure 3b). Even a higher level of expression (10-fold) of this enzyme in *Sb. thermotolerans* cells that were grown in the presence of high amounts of zinc in the medium, which contained no sulfur substrates (our unpublished data), suggests a possible role of SQR in the metal resistance mechanism, in addition to its role in the sulfur metabolism. A hypothetical protein (BXT84_01950, Table 3), which is encoded by the gene located upstream of the SQR gene (BXT84_01945) in the *Sb. thermotolerans* genome, was also upregulated (2.2-fold). Its function remains unknown.

The S-adenosylmethionine synthetase was also shown to be regulated in *Sb. thermotolerans*. This enzyme was represented by two protein spots both of which were upregulated (2.4- and 5.0-fold). Overexpression of the S-adenosylmethionine synthetase that plays a pivotal role in the central metabolism of all organisms is expected to produce increased levels of S-adenosylmethionine. The latter is a precursor for the synthesis of polyamines, including spermidine, which is involved in thiol metabolism, and a metabolite in the trans-sulfuration pathway to cysteine. S-adenosylmethionine is the primary methyl group donor in methylation reactions; it regulates cell signaling, gene expression, and metabolic pathways [77]. The upregulation of this enzyme (3.7-fold) has been found in the cells of *At. caldus* (another member of communities of ACMs) grown in the presence of 200 mM ZnSO_4_ [28]. In the cells of Sb^3+^-resistant clinical isolates of *Leishmania panamensis*, the S-adenosylmethionine synthetase is upregulated exclusively, playing a central role in the upstream synthesis of precursors of trypanothione (a key molecule involved in the Sb-resistance in *Leishmania* parasites), indicating the importance of thiol metabolism in resistance to the antimony in *Leishmania* [78]. In plants, the S-adenosylmethionine synthetase plays an essential role in response to stress, including metal stress [79,80].

**Figure 4:**
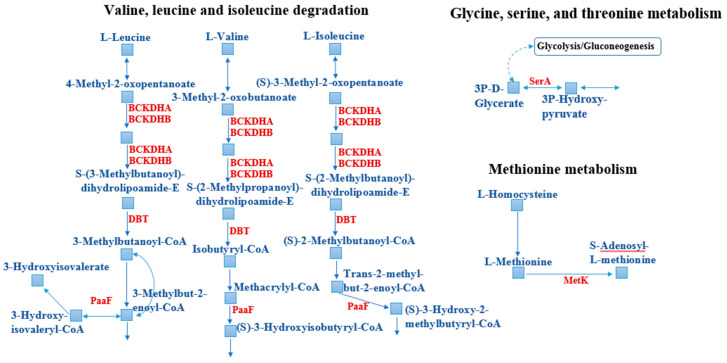
Upregulation of the components of the amino acid metabolism in *Sb. thermotolerans* in response to the gold-bearing pyrite-arsenopyrite concentrate. PaaF: Enoyl-CoA hydratase [EC 4.2.1.17]; DBT: 2-Oxoisovalerate dehydrogenase E2 component (dihydrolipoyl transacylase) [EC 2.3.1.168]; BCKDHB: 2-Oxoisovalerate dehydrogenase E1 component beta subunit [EC 1.2.4.4]; BCKDHA: 2-Oxoisovalerate dehydrogenase E1 component alpha subunit [EC 1.2.4.4]; MetK: S-adenosylmethionine synthetase [EC 2.5.1.6]; SerA: D-3-phosphoglycerate dehydrogenase/2-oxoglutarate reductase [EC 1.1.1.95 1.1.1.399]. Substrates, intermediates, and products are indicated in blue and upregulated enzymes are indicated in red.

Several enzymes participating in amino acid degradation in *Sb. thermotolerans* were also upregulated (Figure 4, Table 3). They included two subunits of the 2-oxoisovalerate dehydrogenase enzyme, which is involved in valine, leucine, and isoleucine degradation, as well as propionate metabolism. The upregulation of this protein has been found in *Sphingomonas* sp. cells in response to Cd^2+^ [70]. Other enzymes of amino acid metabolism were also upregulated, including cysteine desulfurase (5.4-fold; it catalyzes the removal of elemental sulfur and selenium atoms from L-cysteine, L-cystine, L-selenocysteine, and L-selenocystine to produce L-alanine) and D-3-phosphoglycerate dehydrogenase (2-fold). Amino acids are known to be potential ligands for heavy metals and contribute to tolerance and detoxification [66].

The general response of *Sb. thermotolerans* Kr1 cells involved also changes in the transcription, translation, cell biogenesis, coenzyme transport and metabolism, as well as the metabolism of fatty acids and secondary metabolites (Figure 2, Table 3). For instance, the YebC/PmpR family DNA-binding transcriptional regulator was found to be upregulated (2-fold) in response to the ore concentrate. The YebC family regulator proteins are widespread and conserved in many bacteria. YebC may serve as a multi-functional transcription regulator; it is predicted to regulate the resolvase complex RuvABC, most likely the RuvC subunit, and may participate in other biological processes [81]. The YebC protein is responsible for the quorum-sensing (QS) and virulence in *Edwardsiella piscicida,* the negative regulation of the QS response regulator in *Pseudomonas aeruginosa*, and, probably, proteolysis in *Lactobacillus* [82]. In the metal-tolerant bacterium *Alishewanella* sp., the YebC family protein RuvR is involved in Cr^6+^, As^3+^, Sb^3+^, and Cd^2+^ resistance [83]. The NusA protein that participates in transcriptional elongation, termination, anti-termination, as well as cold shock and stress-induced mutagenesis [84], was also upregulated (2.2-fold) in response to the arsenic-rich sulfide concentrate. NusA is suggested to serve as a molecular chaperone in addition to its functions as a transcription factor [84]. In the case of *Sb. thermotolerans*, the upregulation of this protein could also be associated with the stress effect of toxic compounds in the medium of growth.

Thus, on the basis of differential proteomic analysis, a total of 33 proteins were regulated in *Sb. thermotolerans* in response to the high-arsenic sulfide concentrate. Although higher numbers of regulated proteins may be expected under metal(loid) stress [23], the results of our study are in agreement with the previously reported proteomic data for other acidophiles. For instance, in *At. ferrooxidans*, *At. caldus, Am. ferrooxidans*, and *F. acidarmanus*, 7–21 proteins were upregulated and 1–22 proteins were downregulated in response to zinc, arsenic, copper, and uranium stress [24,26,28,85].

It should be mentioned that mainly planktonic late-exponential vegetative cells (already detached or not yet attached to the mineral particles) and single forespores (~1–1.5%) from the liquid phase, which contained iron, arsenic, and other dissolved elements, were analyzed in this study. According to other proteomic reports, differences in the proteomic responses of the planktonic and sessile (biofilm) cell subpopulations of *At. ferrooxidans*, oxidizing pyrite, have been revealed [37,41]. Therefore, the accumulation of elements by sessile cells of *Sb. thermotolerans* and their proteomic response could vary from those of the planktonic cell population.

Overall, the results of our research imply that changes in the protein expression in *Sb. thermotolerans* cells were closely related to the accumulation of the toxic arsenic. Several proteins that seem to confer resistance to arsenic in other microorganisms [66,73,76,83,84], but do not belong to any specific arsenic-resistance systems, were shown to be upregulated in this acidophilic bacterium. In other studied acidophiles, responses to arsenic are associated with specific arsenic resistance proteins [31,33,34]. The latter, as well as the proteins involved in phosphate metabolism, protection from reactive oxygen species, GSH metabolism, DNA synthesis and repair, protein synthesis, folding and refolding, are regulated in *L. ferriphilum* in response to the arsenic stress [33]. In acidophilic archaeon *F. acidarmanus*, the proteomic analysis has not detected increased expression of the ArsB pump; the proteomic response to arsenic involves a number of protein repair and modification enzymes, as well as metabolic and electron transport proteins [85]. However, few studies on the proteomic responses of acidophiles to arsenic have been reported in the literature [33,85], and future proteomic research would be of interest to provide a better understanding of the molecular mechanisms for arsenic resistance in this microbial group.

It is noteworthy that according to our proteomic data on the metabolic reorganization, no pathways were inhibited in *Sb. thermotolerans* in response to the high-arsenic sulfide concentrate. On the contrary, some pathways were reinforced, while iron oxidation rates remained rather high. These results suggest that the components of carbon, energy, and amino acid metabolism, as well as those of stress response systems, may be essential in the resistance mechanisms of *Sb. thermotolerans* to the toxic effects of the gold-containing pyrite-arsenopyrite concentrates. The efficient iron oxidation and reinforcement of catabolic pathways indicate the advantages of the mixotrophic lifestyle of *Sb. thermotolerans* during adaptation to stress factors. This trophic strategy makes it possible to meet the increased metabolic demands of the bacterium under adverse conditions and enhance its growth. Due to the utilization of organic compounds, *Sb. thermotolerans* may also play an essential role in the detoxification of the environment for chemolithoautotrophs (removal of microbial metabolites and lysis products) in acidophilic microbial communities exposed to metal(loid) stress.

## 4. Conclusions

The results of our research provide first insights into the mechanisms of resistance to the gold-bearing arsenopyrite concentrates in the genus *Sulfobacillus*. It was shown that:

- *Sb. thermotolerans* rapidly adapted to the toxic amount of the high-arsenic gold-bearing sulfide concentrate and proved to be among the most As-tolerant organisms known to date;
- although the cellular adsorption and intracellular accumulation of metal(loid)s occurred to high levels, the bacterium retained its growth and efficient substrate oxidation;
- in total, 30 upregulated proteins were involved in the response to the toxic content of the sulfide concentrate in the growth medium, and only three proteins, which, however, did not affect the vital activity of the preliminarily nonadapted *Sb*. *thermotolerans* cells*,* were downregulated;
- *Sb. thermotolerans* cells responded to adverse conditions by metabolic changes, including reinforcement of pathways of constructive and energy metabolism, and by activation of defense systems against unfavorable factors. At the same time, no specific metal-resistance components were regulated in response to metal(loid)s accumulated in the culture medium and by the cells;
- proteins of the stress response, such as the metal-related stress protein MBL fold metallo-hydrolase and GroEL chaperonin, probably, played crucial roles in the tolerance to the high-arsenic sulfide ore concentrate and arsenic, in particular;
- the markedly upregulated sulfide:quinone oxidoreductase, cysteine desulfurase, and S-adenosylmethionine synthetase were other main contributors to the bacterial response. Apart from the enzymatic function in sulfur metabolism, sulfide:quinone oxidoreductase potentially fulfilled the second function of a resistance-conferring protein in *Sb. thermotolerans*.

This study and previous works indicate a high resistance potential of *Sb. thermotolerans*, which is of interest to both the fundamental science and industrial applications. Our results may open up new perspectives on the investigation of the roles of certain proteins in the tolerance mechanisms to different metal(loid)s in *Sulfobacillus* bacteria and other biomining acidophiles.

## Figures and Tables

**Table 1:** Contents of the main elements in the original sulfide concentrate and concentrations of metal(loid)s in the liquid phase after 80 h of cultivation with *Sb. thermotolerans* Kr1.

Element		Content (wt%),Original Concentrate	Element Concentration (mM), Liquid Phase (after 80 h)
Fe	Fe_tot_^a^	38.7 ± 0.51	39.0 ± 2.2
	Fe_S_^b^	34.9 ± 0.89	
S	S_tot_	28.25 ± 0.76	27.1 ± 1.5
	S_S_	28.1 ± 0.95	
As	As_tot_	19.1± 0.75	35.3 ± 3.7 (4.4 As^5+^ and 30.9 As^3+^)
	As_S_	16 ± 0.15	
Sb		0.15 ± 0.03	nd ^c^ (˂0.550)
Zn		0.72 ± 0.02	1.5 ± 0.1
Cu		0.04 ± 0.01	nd (˂0.077)
Pb		1.21 ± 0.06	nd (˂0.190)
Au		135.2 ± 1.61 g/t	− ^d^
Ag		160 ± 2.35 g/t	−

^a^ Tot: Total; ^b^ The index *s* indicates the content of the element in sulfidic minerals; ^c^ nd: Not detected (the value is below the test-sensitivity level, which is shown in parentheses); ^d^ Au and Ag are in sulfides. Mean values ± SD (*p* ≤ 0.05) are shown.

**Table 2:** Total cellular accumulation and intracellular concentrations of metal(loid)s at the late-exponential phase of *Sb. thermotolerans* growth.

Element	Growth Conditions			
	Optimal		Ore Concentrate, 20 g/L	
	C_tot_, µg/g ^a^	C_intra_, µg/g ^b^	C_tot_, µg/g ^a^	C_intra_, µg/g ^b^
Fe	60210 ± 350	830 ± 4.9	6515 ± 59	588 ± 6
Zn	53.5 ± 1.5	31.7 ± 2.5	1338 ± 74	463 ± 5.9
Cu	29 ± 0.54	16 ± 0.7	165.5 ± 18	125 ± 7
Pb	2.85 ± 0.36	<0.00098 ^c^	370 ± 16.7	74 ± 2.7
Ag	1.35 ± 0.25	<0.066 ^c^	135.5 ± 6.4	10 ± 1.4
Au	0.027 ± 0.003	<0.0017 ^c^	11.8 ± 0.6	9.9 ± 0.5
As	1.75 ± 0.07	0.94 ± 0.17	941 ± 46	233.5 ± 6
Sb	1.7 ± 0.1	0.33 ± 0.1	53.5 ± 5.7	26.5 ± 2.3

^a^ C_tot_ and ^b^ C_intra_: Total cellular and intracellular element concentration, respectively (µg per g dried biomass); ^c^ the value is below the test-sensitivity level. Mean values ± SD (*p* ≤ 0.05) are shown.

**Table 3:** Proteome reorganization in the *Sb. thermotolerans* cells grown under optimal conditions (Cy5, red) and in the presence of the gold-bearing arsenopyrite concentrate (Cy3, green). Protein spot numbers and ratios in green or red indicate up- or downregulation, respectively, in response to the sulfide concentrate.

Protein Spot No. ^a^	Putative Homolog	Protein ID, Size (a. a.)	Function(s)	Cy3/Cy5 Ratio
1	MBL fold metallo-hydrolase	BXT84_14770, 312	Hydrolytic enzymes that include class B β-lactamases, hydroxyacylglutathione hydrolases, persulfide dioxygenases, flavodiiron proteins, insecticide hydrolases.	6.3 ± 0.82
2	Sulfide:quinone oxidoreductase [EC 1.8.5.4]	BXT84_01945, 379	Reaction of transformation of H_2_S to polysulfide, in which two electrons are transferred to the electron chain by quinone. *Energy production and conversion. Sulfur metabolism.*	5.8 ± 0.75
3	Cysteine desulfurase	BXT84_05690, 406	Removal of elemental sulfur and selenium atoms from L-cysteine, L-cystine, L-selenocysteine, and L-selenocystine to produce L-alanine. *Amino acid transport, metabolism, and degradation.* *Biosynthesis of cofactors, prosthetic groups, and carriers.*	5.4 ± 0.70
4	Chaperonin GroEL	BXT84_08625, 548	Productive folding of proteins. *Chaperones, chaperonins, stress proteins, etc.*	5.3 ± 0.69
5	S-adenosylmethionine synthetase [EC 2.5.1.6]	BXT84_12950, 399	DNA methylation. Control of gene expression. *Coenzyme transport and metabolism. Gene transcription. Cell proliferation.*	5.0 ± 0.65
6	2-Oxoisovalerate dehydrogenase subunit alpha	BXT84_00985, 331	Oxidative decarboxylation of 4-methyl-2-oxopentanoate, 3-methyl-2-oxopentanoate, and 3-methyl-2-oxobutanoate. *Amino acid transport, metabolism, and degradation.*	3.2 ± 0.42
7	Triose-phosphate isomerase	BXT84_02930, 241	Interconversion of dihydroxyacetone phosphate and D-glyceraldehyde-3-phosphate. *Energy production and conversion. Carbon metabolism. Glycolysis/gluconeogenesis.*	3.2 ± 0.42
8	Branched-chain α-keto acid dehydrogenase subunit E2	BXT84_00995, 436	Pyruvate/2-oxoglutarate dehydrogenase complex, dihydrolipoamide acyltransferase (E2) component. TCA cycle and valine, leucine, and isoleucine degradation. *Energy production and conversion. Carbon metabolism. Amino acid transport, metabolism, and degradation.*	3.1 ± 0.40
9	Hydroxypyruvate isomerase	BXT84_05190, 259	Interconvertion of hydroxypyruvate and 2-hydroxy-3-oxopropanoate. *Glyoxylate and dicarboxylate metabolism. Carbohydrate transport and metabolism.*	3.1 ± 0.40
10	Peptidase C26Protein MinD	BXT84_08510, 247	Gamma-Glutamyl bond cleavage in poly-gamma-glutamyl substrates. *Amino acid transport/metabolism.*	2.6 ± 0.34
		BXT84_03685, 265	MinD stimulates activity of the division inhibitor MinC. *Cell biogenesis, cell division*.	
11	2-Oxoisovalerate dehydrogenase subunit alpha	BXT84_00985, 331	Oxidative decarboxylation of 4-methyl-2-oxopentanoate, 3-methyl-2-oxopentanoate, and 3-methyl-2-oxobutanoate. *Amino acid transport, metabolism, and degradation.*	2.6 ± 0.34
12	S-adenosylmethionine synthetase [EC 2.5.1.6]	BXT84_12950, 399	DNA methylation. Control of gene expression. *Coenzyme transport and metabolism. Gene transcription. Cell proliferation.*	2.4 ± 0.31
13	Voltage-gated K channel	BXT84_12450, 317	Binds NADPH and couples voltage-gated channel activity to the redox potential of the cell.	2.4 ± 0.31
14	Aconitate hydratase [EC 4.2.1.3]	BXT84_12440, 900	The reversible isomerization of citrate/isocitrate (cis-2-methylaconitate/2-methylisocitrate) in the TCA/methylcitrate cycle. *Energy production and conversion.* *Carbon metabolism.*	2.4 ± 0.31
15	Rubrerythrin	BXT84_08520, 140	Reduction of H_2_O_2_ (oxidative stress protection system). *Energy production and conversion.*	2.3 ± 0.29
16	Thioredoxin peroxidase (peroxiredoxin)	BXT84_15685, 178	Homodimeric thiol-specific antioxidant protein reducing and detoxifying H_2_O_2_, peroxynitrite, and organic hydroperoxides. *Detoxification. Oxidative stress defense.*	2.3 ± 0.29
17	Enoyl-CoA hydratase	BXT84_12400, 254	An important role in fatty acid metabolism. *Lipid transport and metabolism.*	2.3 ± 0.30
18	Peptide ABC transporter substrate-binding protein	BXT84_06635, 565	ABC-type transport system. Involved in the transport of leucine, isoleucine, and valine. *Amino acid transport, metabolism, and degradation.*	2.3 ± 0.30
19	1,4-dihydroxy-2-naphthoyl-CoA synthase [EC 4.1.3.36]	BXT84_09740, 273	Conversion of 2-succinylbenzoate into 1,4-di-hydroxy-2-naphthoate. *Coenzyme transport and metabolism. Biosynthesis of cofactors, prosthetic groups, and carriers.*	2.2 ± 0.28
20	Hypothetical protein	BXT84_01950, 335	DUF1641 domain-containing uncharacterized conserved protein. Function unknown.	2.2 ± 0.28
21	NusA	BXT84_06895, 349	N utilization substance protein A, a bacterial transcription termination factor. It may serve as a molecular chaperone. *Transcription. Transcription factors.*	2.2 ± 0.29
22	Pyridine nucleotide-disulfide oxidoreductase	BXT84_04315, 420	Class Ⅰ and Ⅱ oxidoreductases and NADH oxidases and peroxidases. *Energy production and conversion.*	2.2 ± 0.29
23	Glucose-6-phosphate 1-dehydrogenase	BXT84_09125, 513	D-glucose 6-phosphate + NADP^+^ ↔ 6-phospho-D-glucono-1,5-lactone + NADPH + H^+^. *Carbon metabolism. Carbohydrate transport and metabolism.*	2.2 ± 0.29
24	2-Oxoisovalerate dehydrogenase subunit beta	BXT84_00990, 327	Oxidative decarboxylation of 4-methyl-2-oxopentanoate, 3-methyl-2-oxopentanoate, and 3-methyl-2-oxobutanoate. *Amino acid transport, metabolism, and degradation.*	2.0 ± 0.26
	ADP-ribosyl-glycohydrolase	BXT84_07905, 340	ADP-ribosylations. *Posttranslational modification, protein turnover, chaperones.*	
25	Succinate-CoA ligase subunit beta	BXT84_09905, 370	Succinate-CoA ligase catalyzes the reversible reaction of succinyl-CoA to succinate. *Energy metabolism. TCA cycle.*	2.0 ± 0.26
	YebC/PmpR family transcriptional regulator	BXT84_10200, 248	Regulation of RuvABC and participation in other biological processes. *Transcription, translation, ribosomal structure, and biogenesis. Regulatory functions, DNA interactions*.	
26	Phosphoglycerate dehydrogenase	BXT84_08460, 519	Biosynthesis of L-serine from D-3-phosphoglycerate. *Amino acid biosynthesis.*	2.0 ± 0.26
27	ATP-dependent Clp protease ATP-binding subunit ClpC	BXT84_02130, 821	ClpC ATPase of the Hsp100 family, a positive regulator of the heat shock response. Molecular chaperon. *Posttranslational modification, protein turnover, chaperones.*	1.9 ± 0.25
28	Chaperonin GroEL	BXT84_08625, 548	Productive folding of proteins. *Chaperones, chaperonins, stress proteins, etc.*	0.5 ± 0.06
29				0.5 ± 0.06
30	Trigger factor	BXT84_03590, 437	A ribosome-associated molecular chaperone. *Protein fate, protein folding, and stabilization.*	0.4 ± 0.05

^a^ Numbers of the protein spots correspond to the numbers in Figure 1b.

## Supplementary Materials

The following are available online at https://www.mdpi.com/2076-2607/8/7/1076/s1. Figure S1: Growth of *Sb. thermotolerans* Kr1 and iron oxidation under different cultivation conditions: (**a**) In the medium containing ferrous iron (control); in the presence of 10 (**b**), 20 (**c**), and 30 g/L (**d**) of the gold-containing pyrite-arsenopyrite concentrate. Arrows (**b**,**d**) indicate parameters of the samples used in the experiments.
