# Supplementary material for: Understanding Stress Response to High-Arsenic Gold-Bearing Sulfide Concentrate in Extremely Metal-Resistant Acidophile Sulfobacillus thermotolerans
Source: Microorganisms. 2020 Jul 19;8(7):1076. doi: 10.3390/microorganisms8071076 (PMC7409299; doi:10.3390/microorganisms8071076)
Supplement: Supplementary file 1 [file microorganisms-08-01076-s001.pdf]

Supplementary Materials

Understanding Stress Response to High-Arsenic Gold-Bearing Sulfide Concentrate in Extremely Metal-Resistant Acidophile *Sulfobacillus thermotolerans*

Anna Panyushkina, Daria Matyushkina and Olga Pobeguts

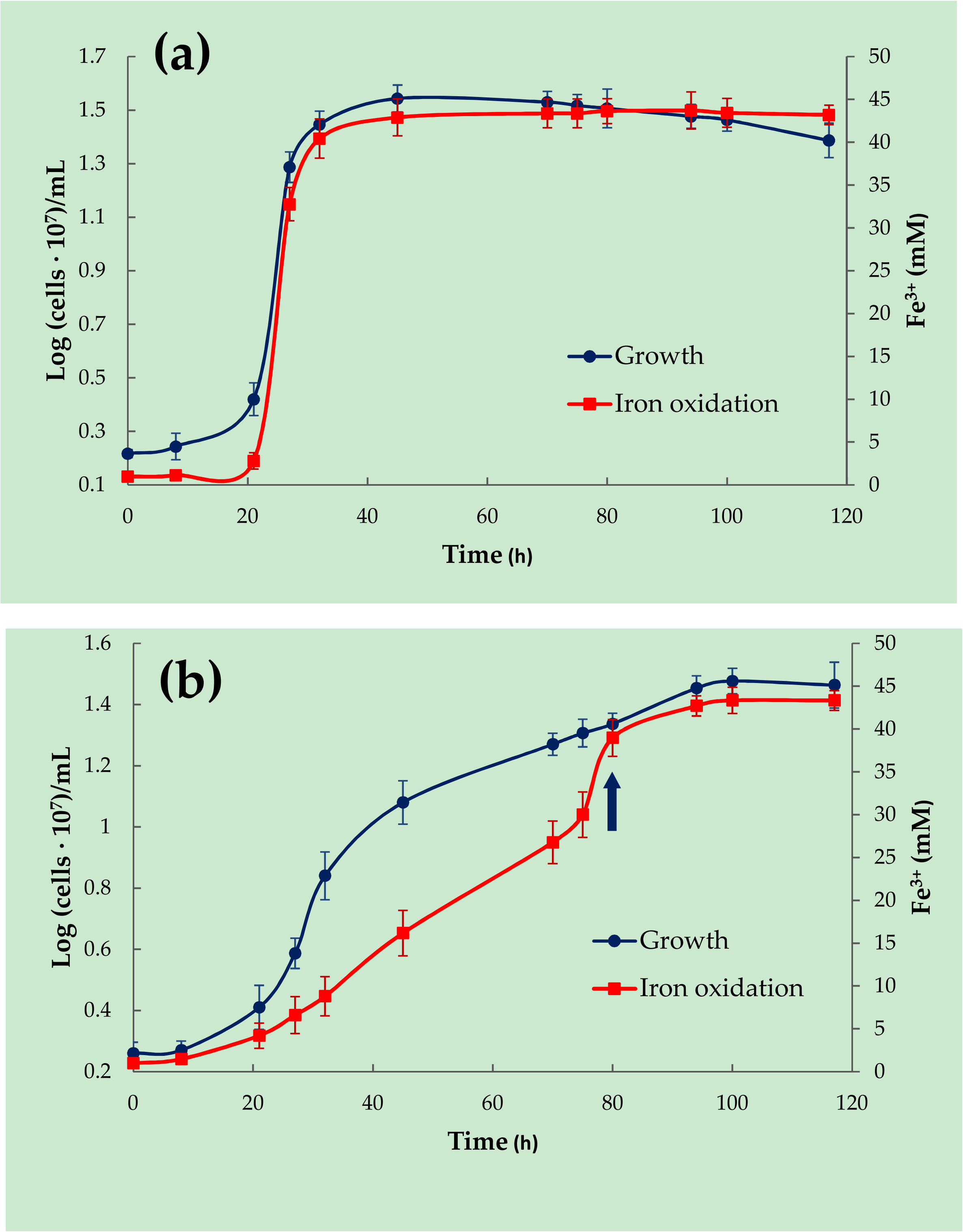

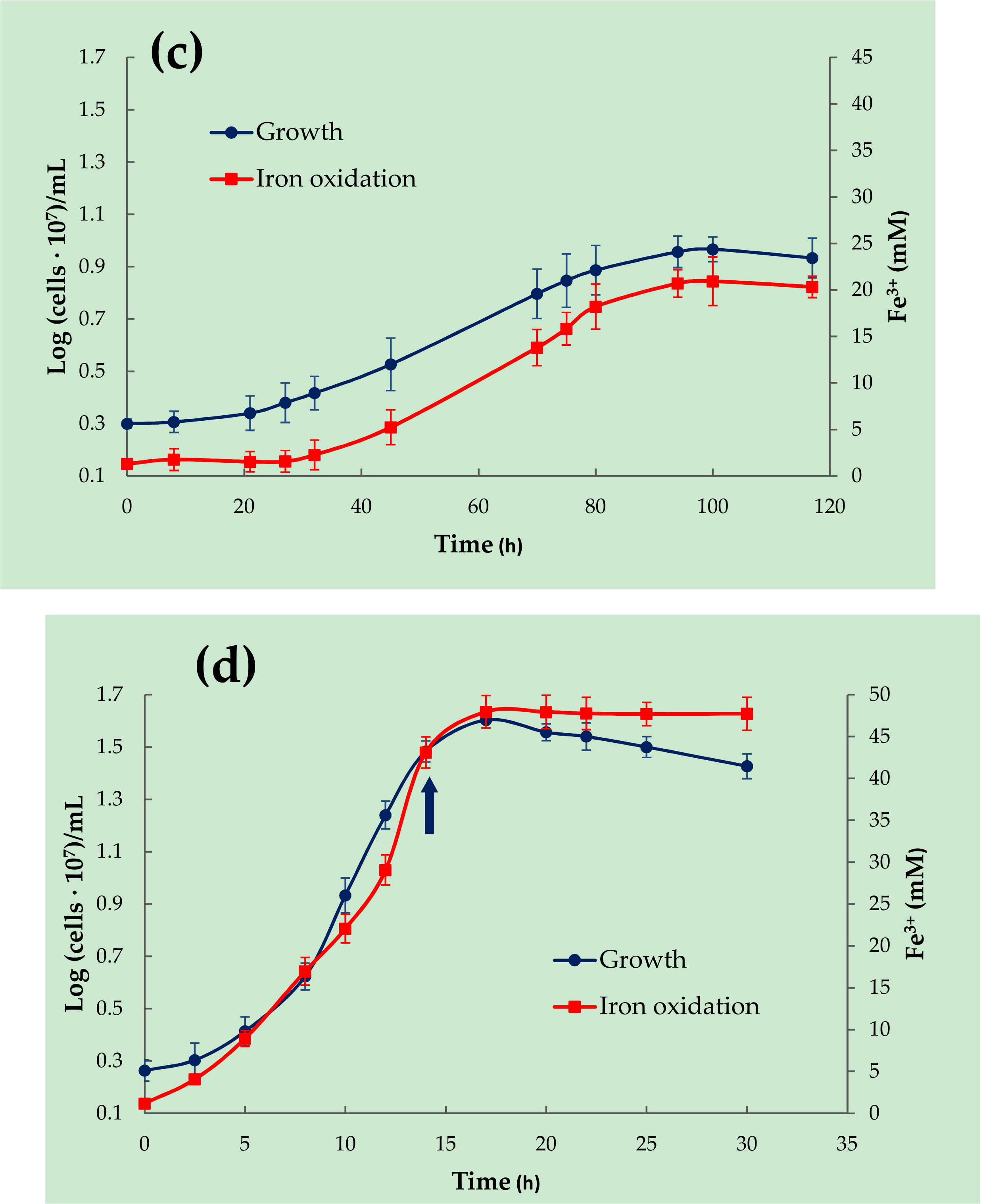

Figure S1. Growth of *Sb. thermotolerans* Kr1 and iron oxidation under different cultivation conditions: (**a**) in the medium containing ferrous iron (control); in the presence of 10 g/L (**b**), 20 g/L (**c**), and 30 g/L (**d**) of the gold-containing pyrite-arsenopyrite concentrate. Arrows (**b**,**d**) indicate parameters of the samples used in experiments.
